# Comparing the Outcomes of Matched and Mismatched Unrelated Allogeneic Hematopoietic Stem Cell Transplantation with Different Anti-Thymocyte Globulin Formulations: A Retrospective, Double-Centre Experience on Behalf of the Polish Adult Leukemia Group

**DOI:** 10.3390/cancers16101891

**Published:** 2024-05-16

**Authors:** Ugo Giordano, Monika Mordak-Domagała, Małgorzata Sobczyk-Kruszelnicka, Sebastian Giebel, Lidia Gil, Krzysztof D. Dudek, Jarosław Dybko

**Affiliations:** 1 Department and Clinic of Endocrinology, Diabetes and Isotope Therapy, 50-367 Wroclaw, Poland; 2 Lower Silesian Center of Oncology, Pulmonology and Hematology, 53-439 Wroclaw, Poland; mordak@dctk.wroc.pl (M.M.-D.); jaroslaw.dybko@dcopih.pl (J.D.); 3 Maria Sklodowska-Curie National Research Institute of Oncology, 44-102 Gliwice, Poland; malgorzata.sobczyk-kruszelnicka@io.gliwice.pl (M.S.-K.); sebastian.giebel@io.gliwice.pl (S.G.); 4 Department of Hematology and Bone Marrow Transplantation, Poznan University of Medical Sciences, 61-701 Poznań, Poland; lidia.gil@skpp.edu.pl; 5 Faculty of Mechanical Engineering, Wroclaw University of Science and Technology, 50-370 Wroclaw, Poland; krzysztof.dudek@pwr.edu.pl; 6 Department of Oncology and Hematology, Faculty of Medicine, Wroclaw University of Science and Technology, 50-370 Wroclaw, Poland

**Keywords:** allogeneic hematopoietic stem cell transplantation, anti-thymocyte globulin, Thymoglobuline, Grafalon, graft-versus-host disease

## Abstract

**Simple Summary:**

Allogeneic hematopoietic stem cell transplantation (allo-HCT) remains an effective treatment modality in many hematological malignancies. Few studies directly compare rabbit anti-thymocyte globulin (r-ATG) formulations Thymoglobuline (ATG-T) and Grafalon (ATG-G). Our retrospective analysis compared the outcomes in 87 adult allo-HCT patients receiving ATG-T or ATG-G. No significant differences were found in acute graft-versus-host disease (aGvHD) incidence. However, chronic GvHD (cGvHD) was less common with ATG-T (7.5% vs. 38.3%, *p* = 0.001). ATG-T patients had higher cytomegalovirus (CMV) reactivation rates (70% vs. 31.9%, *p* < 0.001) and a shorter time to CMV (<61 days, 77.8% vs. 33.3%, *p* = 0.008), but were mostly asymptomatic (85.7% vs. 43.8%, *p* = 0.005). Overall survival (OS) and relapse-free survival (RFS) at 5 and 3 years, respectively, showed no significant differences between ATG-T and ATG-G (32.0% vs. 40.3%, *p* = 0.423; 66.7% vs. 60.4%, *p* = 0.544).

**Abstract:**

Despite notable advancements in immunotherapy in the past decades, allogeneic hematopoietic stem cell transplantation (allo-HCT) remains a promising, potentially curative treatment modality. Only a limited number of studies have performed a direct comparison of two prevalent rabbit anti-thymocyte globulin (r-ATG) formulations—specifically, Thymoglobuline (ATG-T, formerly Genzyme) and Grafalon (ATG-G, formerly Fresenius). The primary objective of our retrospective analysis was to compare the outcomes of adult patients undergoing matched or mismatched unrelated donor (MUD/MMUD) allo-HCT, with a graft-versus-host disease (GvHD) prophylaxis based on either ATG-T or ATG-G. A total of 87 patients who had undergone allo-HCT between 2012 and 2022 were included. We observed no significant differences between ATG-T and ATG-G concerning the occurrence of acute graft-versus-host disease (aGvHD), regardless of its severity. Conversely, chronic graft-versus-host disease (cGvHD) occurred less frequently in the ATG-T group compared to the ATG-G group (7.5% vs. 38.3%, *p* = 0.001). The negative impact of ATG-G on cGvHD was confirmed by multivariate analysis (HR 8.12, 95% CI 2.06–32.0, *p* = 0.003). Patients treated with ATG-T manifested a higher incidence of cytomegalovirus (CMV) reactivations (70% vs. 31.9%, *p* < 0.001), with a shorter time between transplant and CMV (<61 days, 77.8% vs. 33.3%, *p* = 0.008) and a higher median CMV copy number (1000 vs. 0, *p* = 0.004). Notably, despite a higher occurrence of CMV reactivations in the ATG-T cohort, most patients were asymptomatic compared to ATG-G (85.7% vs. 43.8%, *p* = 0.005). By multivariate analysis, only aGvHD had an influence on CMV reactivations (HR 0.18, 95% CI 0.04–0.75, *p* = 0.019). Finally, we observed no significant differences in terms of 5-year overall survival (OS) and 3-year relapse-free survival (RFS) while comparing ATG-T and ATG-G (32.0% vs. 40.3%, *p* = 0.423; 66.7% vs. 60.4%, *p* = 0.544, respectively).

## 1. Introduction

Graft-versus-host disease (GvHD) represents a significant complication in the context of allogeneic hematopoietic stem cell transplantation (allo-HCT) [1,2], exerting detrimental effects on both the duration and quality of life of transplant recipients [3]. Factors such as the use of peripheral blood stem cells (PBSC), an increased T cell count, and HLA mismatch currently stand as major risk factors for susceptibility to both acute GvHD (aGvHD) and chronic GvHD (cGvHD) [4,5,6,7].

Historically, before the 1980s, the commonly utilized GvHD prophylaxis regimen was methotrexate (MTX) [8], and the incorporation of cyclosporine A (CsA) in the following years led to a substantial reduction in the occurrence of aGvHD grade II-IV, reaching 20%, that is, from 50% to 30% [9]. In spite of significant advancements in the prevention of GvHD, aGvHD is experienced by over 40% of patients receiving allo-HCT, particularly those of older age [10,11]. Even with the inclusion of mycophenolate mofetil (MMF) alongside MTX and CsA, aGvHD grade II-IV persists up to 30% in MRD allo-HCT recipients aged 40 years or older [12]. Hence, there is a need to develop more effective GvHD prophylactic regimens.

In Europe, the predominant therapeutic strategy for preventing GvHD involves standard prophylaxis consisting of calcineurin inhibitors (CNIs), MTX, or MMF, along with one of the available rabbit anti-thymocyte globulins (r-ATGs) for unrelated donor transplantation. In recent years, this approach has been extended to sibling donor allo-HCT as well [13]. Various countries have access to different formulations of ATGs derived from rabbits, horses, or pigs, produced through the inoculation of human thymocytes or human cell lines. The latter, that is, porcine ATG (p-ATG) and horse ATG (h-ATG), are less frequently employed medications in the context of European countries.

There are currently two formulations of rabbit anti-thymocyte globulins (rATGs), both composed of polyclonal IgG derived from hyperimmune sera of rabbits. These IgG antibodies are immunized either with human Jurkat leukemia T-cell line in the case of ATG-G (anti-T-lymphocyte globulin, Grafalon; Neovii, Rapperswil, Switzerland; formerly Fresenius) or with human thymocytes in the case of ATG-T (anti-thymocyte globulin, Thymoglobulin; Sanofi, Paris, France; formerly Genzyme) [14]. Additionally, ATG-T and ATG-G differ in the antigens they target. In comparison to ATG-T, the range of antigens recognized by ATG-G is narrower; for instance, ATG-G includes few or no antibodies targeting HLA-DR, CD3, or CD4 [15,16]. Nevertheless, ATG-G possesses more antibodies against CD107, which is expressed on T cells during degranulation, a process resulting from antigenic stimulation [16]. Conversely, ATG-T addresses antigens expressed on T cells (CD2, CD3, CD4, CD6, CD8), natural killer cells, B cells, dendritic cells, and macrophages, as well as HLA-DR and HLA class 1 [12]. ATG-T also consists of antibodies specifically targeting antigens associated with cellular trafficking and adhesion, along with those implicated in inflammation, apoptosis, and cellular proliferation [12]. Competitive binding experiments have indicated that ATG-T demonstrates higher reactivity and a more potent complement-mediated cytotoxic effect toward peripheral blood mononuclear cells than ATG-G. Furthermore, when equal doses of the two formulations are employed, ATG-T more effectively induces apoptosis of dendritic cells than ATG-G. As a result, GvHD prophylaxis typically involves the administration of higher doses of ATG-G than ATG-T.

The immunological consequences of ATG are also influenced by various factors, including the timing of administration considering the day of transplantation, the cumulative dosage, and the recipient’s lymphocyte count at the time of allo-HCT. Higher doses of rATG, a lower host total lymphocyte count, and closer timing to transplantation can lead to prolonged exposure to ATG after donor T cell infusion [4]. This results in a delayed immune reconstitution [17,18], thereby increasing the potential for vulnerability to infections, relapse, and the development of lymphoproliferative disorders following allo-HCT [19]. Hence, these factors must be taken into consideration when evaluating the outcomes associated with rATG administration [4].

The main aim of our retrospective analysis was to assess the impact of ATG-T and ATG-G on graft-versus-host disease as well as overall survival following allo-HCT in adult patients. Also, considering the molecular differences between these two formulations, we examined the effects on the occurrence of viral reactivations.

## 2. Materials and Methods

### 2.1. Patients

Data were obtained through in-depth analysis of the patients’ documentation in two Polish Adult Leukaemia Group (PALG)-associated centers. The individuals underwent allo-HCT in two PALG-associated centers between 2012 and 2022. Patients included in the analysis are of ≥18 years of age with acute myeloid leukemia (AML), myelodysplastic syndrome (MDS), acute lymphoblastic leukemia (ALL), myeloma, lymphoma and other hematological disorders in first, second, third or fourth complete remission. Patients received PBSC allo-HCT from a MUD or MMUD and were administered a myeloablative (MAC), non-myeloablative (NMA) or reduced-intensity (RIC) conditioning regimen. Standard GvHD prophylaxis was employed, based on MTX with tacrolimus (Tac) or CsA with one of the two available rATG formulations—ATG-T or ATG-G. Of note, letermovir CMV prophylaxis, which is currently regarded as the standard of care, only became widely available in Poland in 2021.

### 2.2. Outcomes

The primary endpoint of our study was acute and chronic GvHD. Secondary endpoints included CMV-related outcomes, OS and RFS. Acute and chronic GvHD were defined following the previously published criteria [20,21]. Relapse was defined as the cytogenetic, molecular or morphologic recurrence of the disease. RFS was defined as the time period from transplantation to disease relapse in alive patients.

### 2.3. Statistical Methods

Nominal and ordinal variables are presented in the contingency tables as numbers (*n*) and percentages (%). Quantitative variables are presented as medians and ranges. The chi-squared test of independence or Fisher’s exact test were used to assess the significance of correlation between two qualitative variables. Survival curves and the Kaplan–Meier procedure were used to assess the effectiveness of treatment. The F-Cox test was used to compare survival curves in the two groups. In multivariate analysis, considering that the variables aGvHD, cGvHD, relapse and CMV reactivation are binary variables, logistic regression was employed. Parameters with a *p* value of <0.20 in univariate analysis were included.

All analyses were performed using the statistical software package Statistica v. 13.3 (TIBCO Software Inc., Palo Alto, CA, USA). A *p*-value of <0.05 was considered to be statistically significant.

## 3. Results

### 3.1. Patient-, Disease-, and Transplantation-Related Characteristics

Patient-, disease-, and transplantation-related characteristics are shown in Table 1, Table 2, Table 3, Table 4 and Table 5. The statistically significant results of multivariate analysis are presented in Table 6. A total of 87 patients underwent mismatched- and matched-unrelated allo-HCT, of whom 40 received ATG-T and 47 ATG-G. The higher number of older patients receiving ATG-G rather than ATG-T depended on its availability at the moment of transplant, and was not based on any clinical criteria. The characteristics of the patients in the two groups were comparable except for the patients’ age (≥55 or <55 years), conditioning regimen (RIC, MAC or NMA), and CMV prophylaxis (aciclovir, valganciclovir, letermovir). The ratios of the applied conditioning regimens (MAC/RIC/NMA) were 82.5%/10%/7.5% for ATG-T, and 53.2%/31.9%/14.9% for ATG-G (*p* = 0.014). There were more patients ≥55 years of age in the ATG-T group compared to ATG-G (82.5% vs. 51.1%, *p* = 0.003), and the median ages were 45 years and 55 years (*p* = 0.065) with female/male ratios of 17/23 and 20/27 (*p* = 0.832), respectively. The median times of follow-up were 27 months (7–42) for the ATG-T group, and 16 months (8–44) for the ATG-G one. The donors were either matched unrelated (MUD) or mismatched unrelated (MMUD) (27.5% vs. 72.5%, respectively for ATG-T; 78.7% vs. 21.3%, respectively for ATG-G). Also, the median donor ages and CD34+ count were comparable among the two cohorts (30 years vs. 33 years, *p* = 0.399; 8.1 × 10^6^/kg vs. 7.4 × 10^6^/kg, *p* = 0.810, respectively), Most individuals in both the ATG-T and ATG-G group were diagnosed with AML or MDS (37.5% vs. 53.2%, respectively), followed by ALL (25% vs. 17%, respectively) and lymphoma/myeloma (20% vs. 14.9%, respectively). Other diagnoses were classified separately, constituting a mere 17.5% and 14.9%.

### 3.2. Acute and Chronic GvHD

**Figure 1:**
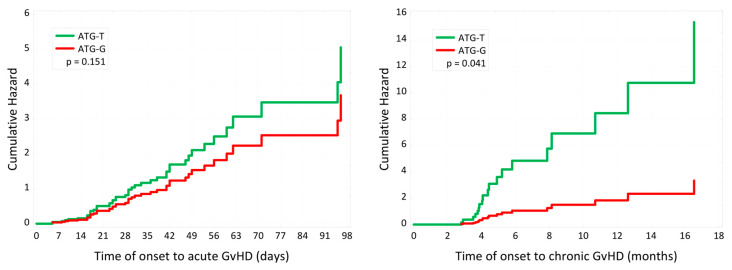
Cumulative hazard of acute GvHD and chronic GvHD with ATG-T and ATG-G.

The results concerning acute and chronic GvHD are presented in Table 3 and Figure 1. No statistically significant discrepancies were observed in the occurrence of aGvHD comparing ATG-T and ATG-G. However, there was a tendency for ATG-T to cause aGvHD, aGvHD grades II–IV more frequently, with a lower median time of onset of aGvHD in contrast to ATG-G (65% vs. 44.7%, *p* = 0.084; 42.5% vs. 31.9%, *p* = 0.308; 24 vs. 29 days, *p* = 0.366, respectively). Chronic GvHD (cGvHD) was significantly less frequent in the ATG-T group compared to that in the ATG-G group (7.5% vs. 38.3%, *p* = 0.001). Multivariate analysis confirmed the adverse impact of ATG-G on cGvHD (HR 8.12, 95% CI 2.06–32.0, *p* = 0.003) and unveiled the influence of positive donor CMV IgG status on aGvHD (HR 0.19, 95% CI 0.05–0.76, *p* = 0.02).

### 3.3. Infection-Related Mortality and Viral Infections

The infectious outcomes are shown in Table 4. Patients treated with ATG-T experienced a higher incidence of CMV reactivations (70% vs. 31.9%, *p* < 0.001), with a shorter time between transplant and the number of virus copies beyond threshold (<61 days, 77.8% vs. 33.3%, *p* = 0.008) and a higher median CMV copy number (1000 vs. 0, *p* = 0.004). The number of patients who received letermovir as CMV prophylaxis was similar between the ATG-T and ATG-G groups (7.5% vs. 10.9%). Interestingly, despite a higher number of CMV reactivations in the ATG-T cohort, there were more asymptomatic patients than in the group administered ATG-G (85.7% vs. 43.8%, *p* = 0.005). By multivariate analysis, only aGvHD had an impact on CMV reactivations (HR 0.18, 95% CI 0.04–0.75, *p* = 0.019). If CMV is not considered, other viral reactivations were more numerous in the ATG g group (66% vs. 15%, *p* = 0.001), with a statistically significant difference in Epstein–Barr virus (EBV), BK virus (BKV), and JC virus (JCV) reactivations (40.4% vs. 10%, *p* = 0.001; 29.8% vs. 5%, *p* = 0.004; 21.3% vs. 2.5%, *p* = 0.010, respectively).

### 3.4. Survival Outcomes

**Figure 2:**
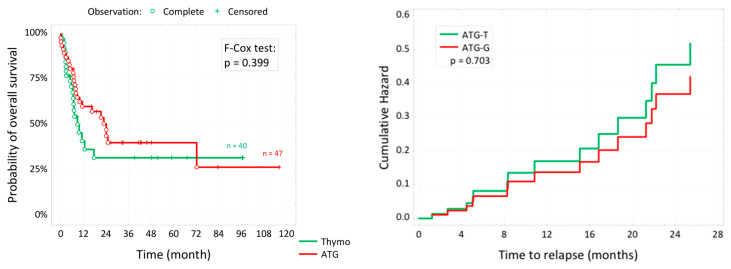
Overall survival and incidence of relapse with ATG-T (Thymo) and ATG-G (ATG).

The survival outcomes are shown in Table 5 and Figure 2. The 5-year overall survival (OS) and 3-year relapse-free survival were comparable between the two cohorts (32.0% vs. 40.3%, *p* = 0.423; 66.7% vs. 60.4%, *p* = 0.544, respectively). By multivariate analysis, NMA conditioning was a predictor of relapse (HR 4.56, 95% CI 1.04–20.3, *p* = 0.045).

## 4. Discussion

Despite important progress made in the area of transplantation procedures, GvHD stands as a major concern limiting allo-HCT’s success, and it is one of the most severe complications [1,2]. Also, GvHD negatively impacts patients’ quality of life and can, in some instances, be fatal [22]. Consequently, endeavors are directed towards identifying the optimal GvHD prophylaxis protocol aimed at GvHD incidence while maintaining a favorable graft-versus-leukemia (GvL) response and minimizing the incidence of potentially lethal viral reactivations. This is of major importance, especially in patients undergoing allo-HCT from mismatched related and unrelated donors with PBSC given their well-established status as predisposing factors for GvHD [4,6]. In this study, we performed a retrospective analysis of patients suffering from various hematological conditions who sustained matched and mismatched unrelated allo-HCT and compared the outcomes of 40 patients who received ATG-T with those of 47 patients who were administered ATG-G.

Our subgroup analysis unveiled no significant difference in the incidence of aGvHD between ATG-T and ATG-G, with a tendency for ATG-T to promote the development of aGvHD, and aGvHD grades II–IV with a lower median time of onset of aGvHD (65% vs. 44.7%, *p* = 0.084; 42.5% vs. 31.9%, *p* = 0.308; 24 vs. 29 days, *p* = 0.366, respectively). Our results are in line with most published studies that carried out a comparison of ATG-T and ATG-G [23,24,25,26,27]. Discrepancies concerning aGvHD grades III–IV can be found in the studies by Oosterbrink et al. [24], comparing ATG-T and ATG-G in MUD/MMUD allo-HCT, and Liu et al. [25], evaluating the outcomes of allo-HCT with ATG-T and ATG-G in mixed donor settings. Their results are contradictory, as the former study demonstrated favorable results for ATG-G (0% vs. 12%, *p* = 0.025) [24], and the latter for ATG-T (2.27% vs. 17.39%, *p* = 0.026) [25]. In terms of cGvHD, we found that the employment of ATG-T rather than ATG-G led to advantageous outcomes (7.5% vs. 38.3%, *p* = 0.001). This finding was confirmed in multivariate analysis as ATG-G was found to be a risk factor for cGvHD (HR 8.12, 95% CI 2.06–32.0, *p* = 0.003). The broad antibody spectrum of ATG-T could be related to its connection with a lower incidence of cGvHD as it is a polyclonal antibody that targets CD19 and CD20, two molecules present on the surface of B cells [28]. Other trials did not demonstrate any relevant discrepancies [23,24,25,26,27]. In contrast, a network meta-analysis by Gagelmann et al. [29] revealed a higher efficacy of ATG-G in preventing cGvHD and aGvHD compared to ATG-T and standard treatment.

It is commonly acknowledged that the use of r-ATG is correlated with CMV reactivation [30], which may result in significant complications following allo-HCT. The use of both ATG-G and ATG-T may delay immune reconstitution, leading to a heightened susceptibility to infections [31,32]. Nevertheless, both formulations selectively bind to diverse antigens present on immune cell surfaces, with ATG-T’s spectrum being much wider, consequently exhibiting a more potent T-cell depletion effect [16,17,18]. Letermovir has demonstrated efficacy in reducing the morbidity and mortality associated with CMV reactivation [33]; however, it has only become widely available in Poland since 2021. As a consequence, few patients received it as CMV prophylaxis (7.5% vs. 10.9%). With regard to viral infections besides CMV, we found that they occurred more frequently in the ATG g cohort than in the ATG-T one (66% vs. 15%, *p* = 0.001), which, however, has no support from other trials [23,24,25,26,27]. Also, most studies did not find significant differences in CMV reactivations between the two rATG formulations [23,24,25,27]. In contrast, the results of our study suggest that patients treated with ATG-T rather than ATG-G are more likely to develop CMV reactivations (70% vs. 31.9%, *p* < 0.001), with a higher median CMV copy number (1000 vs. 0, *p* = 0.004) and a shorter time between transplant and CMV reactivation (<61 days, 77.8% vs. 33.3%, *p* = 0.008). Additionally, most CMV reactivations in the ATG-T cohort were asymptomatic (85.7% vs. 43.8%, *p* = 0.005). By multivariate analysis, the presence of aGvHD was the only significant factor to have an impact on CMV reactivations (HR 0.18, 95% CI 0.04–0.75, *p* = 0.019). A similar outcome, also in an unrelated donor allo-HCT setting, was observed in a trial carried out by Wang et al., where ATG-T was the formulation that prompted CMV reactivations (64.6% vs. 29.9%, *p* < 0.001) [26]. The stronger immunosuppressive effect of ATG-T in contrast to ATG-G could be the reason behind this finding. Lastly, neither the 5-year OS nor 3-year RFS were influenced by the type of r-ATG (32.0% vs. 40.3%, *p* = 0.423; 66.7% vs. 60.4%, *p* = 0.544, respectively). These results are in line with the outcomes of other papers [23,24,25,26,27].

This investigation also has limitations due to its retrospective design. Additionally, the group of individuals included in the analysis is relatively small. Owing to the fact that a substantial number of patients were transplanted nearly 10 years ago when the transplantation standards and viral infection prophylactic regimens were not as refined as nowadays, outcomes such as RFS, OS, and infection reactivations may be suboptimal.

## 5. Conclusions

In conclusion, the results of our study suggest that in case of matched and mismatched unrelated donor allo-HCT, the type of administered r-ATG has an important influence on transplantation outcomes. While different formulations of r-ATG do not appear to impact the occurrence of aGvHD, ATG-T seems to lower the incidence of cGvHD compared to ATG-G. Univariate analysis revealed that CMV reactivations develop more frequently, with a higher CMV copy number and earlier in the post-transplantation period with ATG-T, which was not confirmed by multivariate analysis. Also, multivariate analysis demonstrated that aGvHD was the only factor to have an impact on CMV reactivation incidence in both cohorts. Of note, in the case of ATG-T, there were significantly more CMV asymptomatic reactivations.

Based on our experience and the statistical analyses performed, ATG-T seems to be a more reasonable choice compared to ATG-G considering its positive impact on cGvHD and lack of negative influence on aGvHD and survival outcomes, which could translate into better long-term quality of life for patients who have undergone unrelated allo-HCT. It is yet to be established whether ATG-T could possibly worsen CMV-related outcomes, preferably in double-blind randomized trials with the employment of letermovir, currently regarded as the standard of care. Nevertheless, it is plausible that the development of more effective antiviral medications could limit the risk of infectious complications following allo-HCT despite the employment of GvHD prophylaxis with a stronger T-cell depleting effect.

## Figures and Tables

**Table 1:** Patient- and disease-related characteristics. Statistically significant results are highlighted in bold.

	ATG-T (*n* = 40)		ATG-G (*n* = 47)		*p* Value
Median age, y (range)	45 (35–53)		55 (39–62)		0.065
Age, *n* (%)					**0.003**
<55 years	33	82.5%	24	51.1%	
≥55 years	7	17.5%	23	48.9%	
Sex, *n* (%)					0.832
Male	23	57.5%	27	57.4%	
Female	17	42.5%	20	42.6%	
Diagnosis, *n* (%)					0.524
AML + MDS	15	37.5%	25	53.2%	
ALL	10	25.0%	8	17.0%	
HL + NHL + MM	8	20.0%	7	14.9%	
OMF, CML, SAA, etc.	7	17.5%	7	14.9%	
The advancement of the disease, *n* (%)					0.425
Remission	23	57.5%	32	68.1%	
Active	17	42.5%	15	39.1%	
ELN cytogenetic risk, *n* (%)					0.246
Favorable	3	7.5%	11	23.4%	
Intermediate	9	22.5%	9	19.2%	
Adverse	5	12.5%	4	8.5%	
N/A	23	57.5%	23	48.9%	
Complete remission number, *n* (%)					0.147
0	18	45.0%	14	29.8%	
1	16	40.0%	27	57.4%	
2	6	15.0%	3	6.4%	
3	0	0.0%	2	4.3%	
4	0	2.2%	1	2.1%	
CMV IgG, *n* (%)					0.936
Negative	7	17.5%	9	19.2%	
Positive	33	82.5%	38	80.8%	

**Table 2:** Transplantation-related characteristics. Statistically significant results are highlighted in bold.

	ATG-T (*n* = 40)		ATG-G (*n* = 47)		*p* Value
Median donor age, years (range)	30 (24–39)		33 (25–40)		0.399
Donor age, *n* (%)					0.849
<40 years	30	75.0%	35	74.5%	
≥40 years	10	25.0%	12	25.5%	
Donor sex, *n* (%)					0.145
Male	11	27.5%	6	12.8%	
Female	29	72.5%	41	87.2%	
Locus with a mismatch, *n* (%)					0.221
A	13	44.8%	1	10.0%	
B	2	6.9%	2	20.0%	
C	10	34.5%	5	50.0%	
DQ	4	13.8%	2	20.0%	
Donor status CMV IgG, *n* (%)					0.954
Negative	16	40.0%	18	38.3%	
Positive	24	60.0%	29	61.7%	
CMV prophylaxis, *n* (%)					**0.004**
Aciclovir	36	90.0%	27	58.7%	
Valganciclovir	1	2.5%	4	8.7%	
Aciclovir + Valganciclovir	0	0.0%	10	21.7%	
Letermovir	3	7.5%	5	10.9%	
Conditioning regimen, *n* (%)					**0.014**
RIC	4	10.0%	15	31.9%	
MAC	33	82.5%	25	53.2%	
NMA	3	7.5%	7	14.9%	
Median CD34+ count, ×10^6^/kg (range)	8.1 [5.1–9.6]		7.4 [5.5–9.9]		0.810

**Table 3:** Post-transplantation outcomes—graft-versus-host disease. Statistically significant results are highlighted in bold.

	ATG-T (*n* = 40)		ATG-G (*n* = 47)		*p* Value
Acute GvHD, *n* (%)					0.084
Yes	26	65.0%	21	44.7%	
No	14	35.0%	26	55.3%	
Degree of acute GvHD, *n* (%)					0.103
0	14	35.0%	27	57.4%	
1 or 2	18	45.0%	15	31.9%	
3 or 4	8	20.0%	5	10.6%	
Degree of acute GvHD, *n* (%)					0.308
0 or 1	23	57.5%	32	68.1%	
2 or more	17	42.5%	15	31.9%	
Median time of onset of acute GVHD, days (range)	24 [17–36]		29 [19–49]		0.366
Chronic GvHD, *n* (%)					**0.001**
Yes	3	7.5%	18	38.3%	
No	37	92.5%	29	61.7%	

**Table 4:** Post-transplantation outcomes—infections. Statistically significant results are highlighted in bold.

	ATG-T (*n* = 40)		ATG-G (*n* = 47)		*p* Value
CMV reactivation, *n* (%)					**<0.001**
Yes	28	70.0%	15	31.9%	
No	12	30.0%	32	68.1%	
Median time between transplant and CMV reactivation, days (range)	35 [26–51]		69 [33–127]		0.057
Time between transplant and CMV, *n* (%)	*n* = 27		*n* = 15		**0.008**
<61 days	21	77.8%	5	33.3%	
≥61 days	6	22.2%	10	66.7%	
Median CMV copy number (PCR), count (range)	1000 (0–18,000)		0 (0–634)		**0.004**
CMV copy (CR), *n* (%)					**<0.001**
<400 copies	14	35.0%	35	74.5%	
≥400 copies	26	65.0%	12	25.5%	
Symptoms of CMV disease, *n* (%)	*n* = 35		*n* = 16		**0.005**
Yes	5	14.3%	9	56.2%	
No	30	85.7%	7	43.8%	
Disease manifestation, *n* (%)					0.367
No	38	96.0%	37	80.4%	
Pneumonia CMV	0	0.0%	2	4.3%	
Hepatitis	0	0.0%	1	2.2%	
Gastrointestinal	1	1.2%	4	8.7%	
Pancytopenia	0	0.0%	1	2.2%	
Klebsiella	1	1.2%	1	2.2%	
Other viral reactivations:					**0.001**
Yes	6	15.0%	31	66.0%	
No	34	85.0%	16	34.0%	
EBV	4	10.0%	19	40.4%	**0.001**
BKV	2	5.0%	14	29.8%	**0.004**
JCV	1	2.5%	10	21.3%	**0.010**
HHV6	0	0.0%	1	2.1%	1.000
Others	1	2.5%	0	0.0%	0.460

**Table 5:** Post-transplantation outcomes—3-year RFS and 5-year OS.

	ATG-T (*n* = 40)	ATG-G (*n* = 47)	*p* Value
Median months of follow-up (interquartile range) *	8 [5–52]	22 [12–42]	0.110
3-year relapse-free survival RFS (*t* = 3)	66.7%	60.4%	0.544
5-year overall survival *OS* (*t* = 5)	32.0%	40.3%	0.423
Median survival function **	8.9 months	23.1 months	

* Median follow-up time does not include deaths; ** Number of months survived by half the patients.

**Table 6:** Results of multivariate analysis based on patient, disease, and transplant characteristics in both cohorts. Note: only statistically significant results were included. The full results of multivariate analysis are included as Supplementary Materials Tables S1–S4.

Variable	Risk Factor for	HR	95% CI	*p* Value
ATG-G	Chronic GvHD	8.12	2.06–32.0	0.003
Positive donor CMV IgG status	Acute GvHD	0.19	0.05–0.76	0.02
Acute GvHD	CMV reactivation	0.18	0.04–0.75	0.019
NMA conditioning	Relapse	4.56	1.04–20.3	0.045

## Data Availability

The authors confirm that the data supporting the findings of this study are available within this article.

## Supplementary Materials

The following supporting information can be downloaded at: https://www.mdpi.com/article/10.3390/cancers16101891/s1, Table S1: Risk factors for chronic GvHD—Results of univariate and multivariate regression analysis; Table S2: Risk factors for acute GvHD—Results of univariate and multivariate regression analysis; Table S3: Risk factors for CMV reactivation—Results of univariate and multivariate regression analysis; Table S4: Risk factors for relapse—results of univariate and multivariate logistic regression analysis.
