# Supplementary material for: Comparing the Outcomes of Matched and Mismatched Unrelated Allogeneic Hematopoietic Stem Cell Transplantation with Different Anti-Thymocyte Globulin Formulations: A Retrospective, Double-Centre Experience on Behalf of the Polish Adult Leukemia Group
Source: Cancers (Basel). 2024 May 16;16(10):1891. doi: 10.3390/cancers16101891 (PMC11119435; doi:10.3390/cancers16101891)
Supplement: Supplementary file 1 [file cancers-16-01891-s001.zip › cancers-2994099-supplementary.pdf]

Table S1. Risk factors for chronic GvHD - Results of univariate and multivariate regression analysis.

| Risk factors | b | p-value | OR (95% CI) | beta | p-value | OR (95% CI) |
|---|---|---|---|---|---|---|
| ATG-G | 2.035 | 0.003 | 7.66 (2.20-29.1) | 2.094 | 0.003 | 8.12 (2.06-32.0) |
| Age<60 | 0.307 | 0.624 | 1.36 (0.39-4.72) | - | - | - |
| Male gender | 0.511 | 0.333 | 1.67 (0.59-4.73) | - | - | - |
| CMV IgG | -0.806 | 0.177 | 0.45 (0.14-1.45) | -0.895 | 0.174 | 0.41 (0.11-1.50) |
| Conditioning NMA | 0.856 | 0.226 | 2.35 (0.58-9.49) | 0.550 | 0.516 | 1.73 (0.32-9.30) |
| Conditioning MAC | -0.277 | 0.597 | 0.76 (0.27-2.14) | - | - | - |
| Acute GvHD | 0.425 | 0.409 | 1.53 (0.55-4.24) | - | - | - |
| CMV reactivation | -0.348 | 0.492 | 0.71 (0.26-1.93) | - | - | - |
| CMV<400 | -0.856 | 0.118 | 0.42 (0.14-1.25) | -0.278 | 0.674 | 0.76 (0.20-2.81) |
| EBV | 0.396 | 0.489 | 1.49 (0.48-4.61) | - | - | - |
| BKV | 0.446 | 0.466 | 1.56 (0.46-5.25) | - | - | - |
| JCV | -1.273 | 0.242 | 0.28 (0.03-2.40) | -0.592 | 0.642 | 0.55 (0.04-6.90) |
| Reactivation | 0.729 | 0.159 | 2.07 (0.75-5.75) | 0.412 | 0.489 | 1.51 (0.46-4.91) |
| Disease recurrence | 0.856 | 0.226 | 2.35 (0.58-9.49) | 0.091 | 0.909 | 1.10 (0.23-5.29) |

Table S2. Risk factors for acute GvHD - Results of univariate and multivariate regression analysis.

| Risk factors | b | p-value | OR (95% CI) | beta | p-value | OR (95% CI) |
|---|---|---|---|---|---|---|
| ATG-G | -0.833 | 0.063 | 0.43 (0.18-1.05) | -0.694 | 0.169 | 0.50 (0.19-1.35) |
| Age<60 | 0.737 | 0.160 | 2.09 (0.74-5.87) | 0.727 | 0.209 | 2.07 (0.66-6.48) |
| Male gender | -0.383 | 0.385 | 0.68 (0.29-1.63) | - | - | - |
| CMV IgG donor status - positive | -1.550 | 0.026 | 0.21 (0.05-0.82) | -1.676 | 0.020 | 0.19 (0.05-0.76) |
| Conditioning NMA | 0.275 | 0.689 | 1.32 (0.34-5.14) | - | - | - |
| Conditioning MAC | 0.139 | 0.762 | 1.15 (0.46-2.84) | - | - | - |
| Chronic GvHD | 0.425 | 0.409 | 1.53 (0.55-4.24) | - | - | - |
| CMV reactivation | 0.329 | 0.449 | 1.39 (0.59-3.28) | - | - | - |
| CMV<400 | 0.278 | 0.525 | 1.32 (0.56-3.14) | - | - | - |
| The patient is alive | 0.405 | 0.362 | 1.50 (0.62-3.62) | - | - | - |
| EBV | 0.877 | 0.112 | 2.40 (0.81-7.11) | 1.068 | 0.086 | 2.91 (0.86-9.89) |
| BKV | 1.127 | 0.075 | 3.09 (0.89-10.7) | 0.664 | 0.330 | 1.94 (0.51-7.47) |
| JCV | -0.394 | 0.545 | 0.67 (0.19-2.45) | - | - | - |
| Reactivation | 0.243 | 0.575 | 1.28 (0.54-3.01) | - | - | - |
| Disease recurrence | -0.640 | 0.353 | 0.53 (0.14-2.06) | - | - | - |

Table S3. Risk factors for CMV reactivation - Results of univariate and multivariate regression analysis.

| **Risk factors** | **b** | **p-value** | **OR (95% CI)** | **beta** | **p-value** | **OR (95% CI)** |
|---|---|---|---|---|---|---|
| ATG-G | -0.110 | 0.844 | 0.90 (0.30-2.71) | - | - | - |
| Age<60 | 0.136 | 0.833 | 1.15 (0.32-4.12) | - | - | - |
| Male gender | 0.370 | 0.507 | 1.45 (0.48-4.37) | - | - | - |
| Conditioning RIC | -0.985 | 0.105 | 0.37 (0.11-1.23) | -1.128 | 0.125 | 0.32 (0.08-1.38) |
| Conditioning NMA | 0.778 | 0.478 | 2.18 (0.25-19.1) | - | - | - |
| Conditioning MAC | 0.549 | 0.334 | 1.73 (0.56-5.33) | - | - | - |
| Chronic GvHD | -0.806 | 0.177 | 0.45 (0.14-1.45) | -0.674 | 0.303 | 0.51 (0.14-1.86) |
| Acute GvHD | -1.551 | 0.026 | 0.21 (0.05-0.82) | -1.738 | 0.019 | 0.18 (0.04-0.75) |
| CMV reactivation | 1.296 | 0.041 | 3.66 (1.06-12.7) | 0.562 | 0.653 | 1.75 (0.15-21.0) |
| CMV<400 | 1.438 | 0.038 | 4.21 (1.08-16.4) | 1.118 | 0.406 | 3.06 (0.21-43.8) |
| The patient is alive | 0.140 | 0.806 | 1.15 (0.37-3.58) | - | - | - |
| EBV | -0.893 | 0.138 | 0.41 (0.13-1.34) | -0.533 | 0.423 | 0.59 (0.16-2.19) |
| BKV | 0.542 | 0.507 | 1.72 (0.34-8.65) | - | - | - |
| Reactivation | -0.085 | 0.879 | 0.92 (0.31-2.76) | - | - | - |
| Disease recurrence | -0.118 | 0.889 | 0.89 (0.17-4.76) | - | - | - |

Table S4. Risk factors for relapse - results of univariate and multivariate logistic regression analysis.

| Risk factors for relapse | Univariate analisis | | | Multivariate analisis | | |
|---|---|---|---|---|---|---|
| | b | p | OR (95%) | beta | p | OR (95%) |
| ATG-T (yes) | 0.294 | 0.612 | 1.34 (0.43-4.23) | 0.042 | 0.946 | 1.04 (0.30-3.62) |
| Age (years) | 0.010 | 0.654 | 1.01 (0.97-1.05) | -0.001 | 0.980 | 1.00 (0.95-1.05) |
| Male gender (yes) | 0.126 | 0.828 | 1.13 (0.36-3.58) | 0.035 | 0.955 | 1.04 (0.30-3.52) |
| Active disease (yes) | -0.560 | 0.378 | 0.57 (0.16-2.01) | -0.735 | 0.272 | 0.48 (0.13-1.80) |
| CMV IgG (yes) | -0.126 | 0.860 | 0.88 (0.21-3.65) | -0.180 | 0.822 | 0.84 (0.17-4.08) |
| Donor age >40 years | -0.360 | 0.607 | 0.70 (0.17-2.80) | -0.448 | 0.551 | 0.64 (0.14-2.84) |
| Conditioning NMA | 1.386 | 0.059 | 4.00 (0.95-16.8) | **1.525** | **0.045** | **4.56 (1.04-20.3)** |
| EBV | 1.016 | 0.097 | 2.76 (0.83-9.20) | 0.839 | 0.204 | 2.31 (0.63-8.52) |
